# A revision of the trichostrongylid nematode *Cooperia* Ransom, 1907, from deer game: recent integrative research confirms the existence of the ancient host-specific species *Cooperia ventricosa* (Rudolphi, 1809)

**DOI:** 10.3389/fvets.2024.1346417

**Published:** 2024-02-08

**Authors:** Martina Albrechtová, Eva Štefková Kašparová, Iva Langrová, Vlastimil Hart, Birger Neuhaus, Ivana Jankovská, Miroslav Petrtýl, Jan Magdálek, Marta Špakulová

**Affiliations:** ^1^Department of Zoology and Fisheries, Faculty of Agrobiology, Food and Natural Resources, Czech University of Life Science Prague, Prague, Czechia; ^2^Department of Game Management and Wildlife Biology, Faculty of Forestry and Wood Sciences, Czech University of Life Science Prague, Prague, Czechia; ^3^Museum für Naturkunde, Leibniz Institute for Evolution and Biodiversity, Berlin, Germany; ^4^Institute of Parasitology, Slovak Academy of Sciences, Košice, Slovakia

**Keywords:** *Cooperia ventricosa*, *Cooperia pectinata*, deer game, mitochondrial DNA, ribosomal DNA, redescription, gastrointestinal nematodes

## Abstract

The trichostrongylid roundworms of the genus *Cooperia*, which are important in veterinary medicine, currently comprise 19 valid species that parasitize the small intestine of both free-living and domestic ruminants. Only four *Cooperia* spp. have been reported in Europe, namely *C. oncophora, C. punctata, C. curticei* and *C. pectinata*. In 2018–2022, 25 red deer (*Cervus elaphus*) and 30 sika deer (*Cervus nippon*) of both sexes and various ages from several remote locations in the Czech Republic were parasitologically examined. Intestinal nematodes of the genus *Cooperia* were found only in two northern regions. Using the globally recognized key book on trichostrongylid nematodes, they were preliminarily identified as *C. pectinata*. However, a molecular analysis of *cox*2 and ITS rDNA gene sequences revealed that *Cooperia* sp. parasitizing Czech deer is a separate taxon that is more closely related to *C. oncophora* than to *C. pectinata*. A subsequent morphological analysis and literature survey confirmed the independence of deer *Cooperia* sp., which is similar but not identical to bovid *C. pectinata*. Previous long-term correct identifications of bovid *C. pectinata* and misidentifications of deer *Cooperia* species were caused by a fundamental error in the key book mentioned above. Interestingly, the ancient trichostrongylid nematode *Strongylus ventricosus* from the type host red deer (*Cervus elaphus*) shot near Greifswald (Germany) was described by Rudolphi in 1809. Rudolphi's type material (one male and four females) was deposited in the Museum für Naturkunde (Berlin). Later, the ancient species *S. ventricosus* was taken as a synonym for various *Cooperia* spp. Our current re-examination of the type male indicated that there is a relatively good agreement with our new material from Czech deer regarding the most important characteristics of *S. ventricosus* (i.e., the shape and size of the male spicules); however, Rudolphi's type material is in rather poor condition. The suggested resurrection of the deer *Cooperia* sp. in this study as *Cooperia ventricosa* (Rudolphi, 1809) requires verification by collecting and analyzing new nematode material from the type locality near Greifswald.

## 1 Introduction

Nematodes of the genus *Cooperia* Ransom, 1907 (Strongylida, Trichostrongyloidea: Cooperiidae) are gastrointestinal parasites of many wild and domestic ruminants, and some of these nematodes are distributed worldwide (1). There is a rich species spectrum, predominantly in Africa and other tropical and subtropical regions, where up to 40% of cattle and goats are infected with *Cooperia* spp. (2–5). Although *Cooperia* spp. are not highly pathogenic parasites, a high nematode burden can substantially reduce host production, as infestation has been associated with loss of appetite and poor weight gain (6).

*Cooperia* spp. are monoxenous parasites with free-living pre-parasitic larval phases. Adult worms that reside in the small intestine of the ruminant host produce eggs that are passed in the host's feces. The first-stage larvae hatch in the so-called “fecal pat” where they feed on soil/fecal bacteria. Two subsequent molts are completed within 2 days (7, 8). The third-stage larva remains enclosed in the second-stage cuticle sheath and becomes infective to the host in 1–6 weeks (9). The larvae migrate to the grass and can survive for up to 1 year until swallowed by a ruminant host (10). In the host's small intestine, the larvae shed their sheath, undergo the last two molts, and become sexually mature males or females. When the fertilized females produce eggs, the cycle is complete (11, 12). However, under unfavorable environmental conditions, a life-cycle variation can occur that involves a slowing of development. This strategy involves larval L_4_ hypobiosis (developmental restriction) within the host digestive tract for up to several months (13, 14).

The latest taxonomic revision confirmed that there are 19 valid species of the genus *Cooperia* (1). So far, only four congeners have been reported in Europe, namely *C. curticei*, C. *oncophora, C. pectinata*, and *C. punctata*. All of them have been reported almost worldwide in both wild and domestic ruminants [e.g., (15, 16)]. It is not entirely certain that all previous morphological identifications were reliable, as *Cooperia* congeners are morphologically very similar to each other. The only morphological traits with a high discriminatory value are the size and shape of the male spicules, the characteristics (shape, length, and spatial arrangement) of the rays of the male bursa, and partly the synlophe morphology (longitudinal cuticular ridges) [e.g., (17, 18)]. However, even these traits can sometimes be problematic, as has been the case with the *C. pectinata* species.

**Figure 1:**
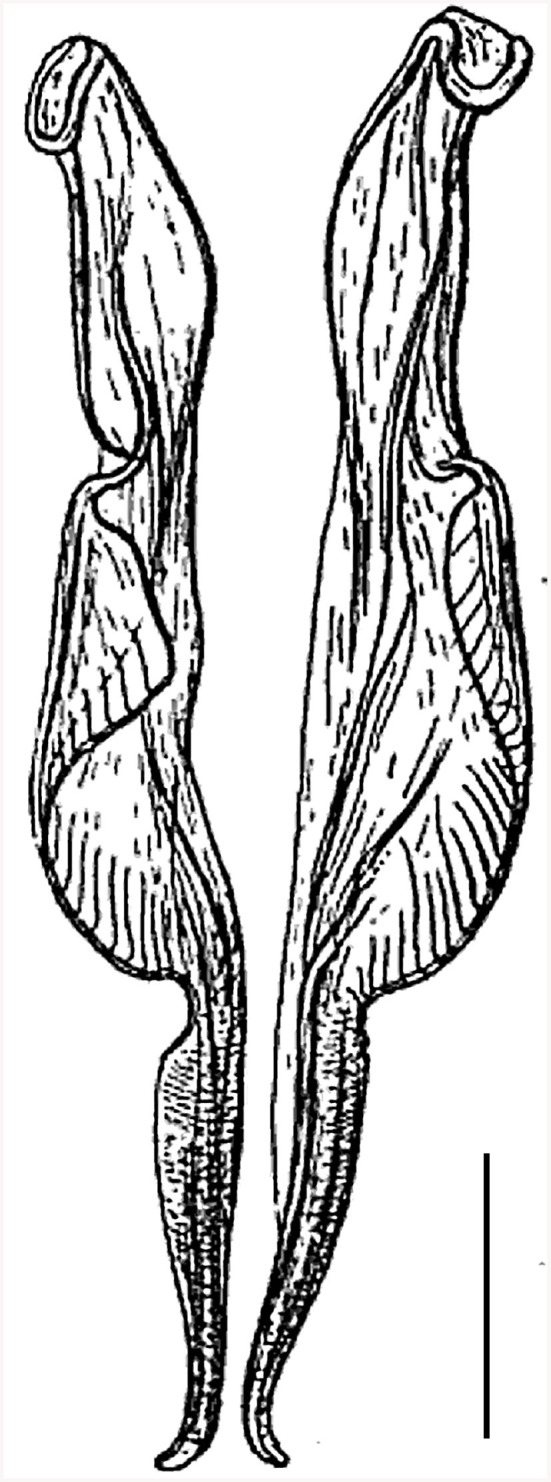
Drawings of spicules of *Cooperia pectinata* Ransom, 1907, which parasitizes bovids (*Bos taurus*), published by Ransom (20). Scale bar = 50 μm.

**Figure 2:**
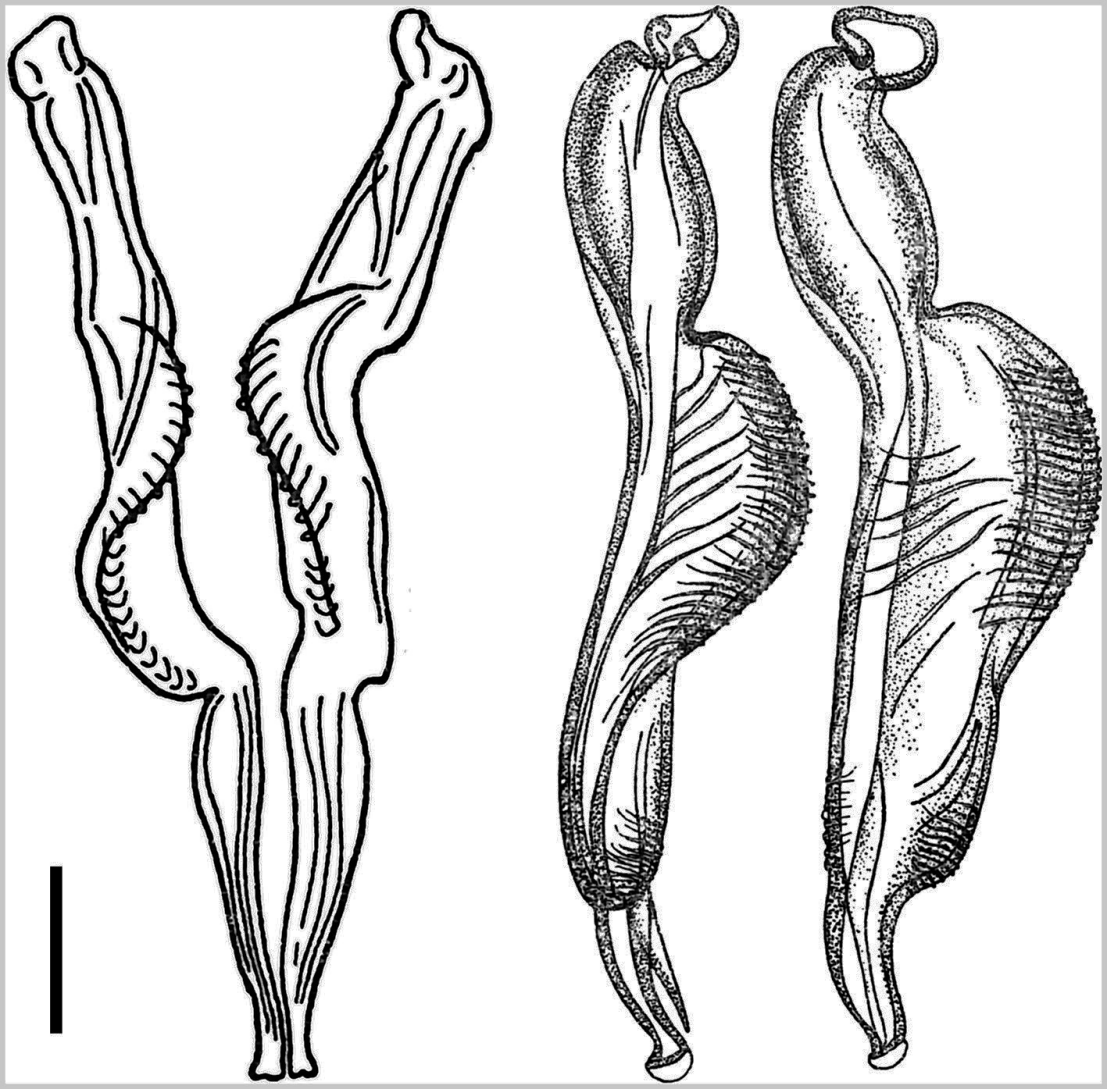
Details of hitherto recognized variants of spicule types of the species *Cooperia pectinata* Ransom, 1907 **(left, right)**, published together in the monograph by Skrjabin et al. (15). According to information by Skrjabin et al. (15), **(left)** is a copy from Baylis (21) and the host species of *C. pectinata* was cattle, while **(right)** was copied from Skrjabin and Orlov (22) and the host of *C. pectinata* was deer. Scale bar = 50 μm.

*Cooperia pectinata* was first described in a paper by Ransom (19) as a parasite of bovids (*Bos taurus*) from Texas, USA. However, this paper only included a short verbal description without any images. Moreover, Ransom noted that “*C. pectinata* might be identical with *Strongylus ventricosus* Rudolphi, 1809 from red deer (*Cervus elaphus*); additionally, the determination of this point necessitates the restudying of the type specimens of Rudolphi”. Later, in 1911, the same author (20) published drawings of the male spicules and bursa, the female vulva, and the exact measurements of *C. pectinata* (Figure 1). After two decades, Baylis (21) published new drawings of *C. pectinata* from Australian cattle, which were later copied into a key Russian monograph on the superfamily Trichostrongyloidea by Skrjabin et al. (15) (Figure 2, left). More recent drawings and photographs are now available of the typical morphological features of *C. pectinata* from cattle and sheared sheep in Brazil (6, 23), from long-term bred alpacas in Australia (24), and from impalas in Africa (16).

However, a confusing incident occurred in the 1930s, when another very similar *Cooperia* nematode was dissected by R. S. Schulz from a sika deer (*Cervus nippon*) in the Russian Far East. This deer *Cooperia* was mistakenly suggested to be *C. pectinata* by I. V. Orlov, who drew the original images of the morphologically important features (22). Although the spicules of these deer nematodes (Figure 2, right) differed slightly from the spicules of the bovid *C. pectinata* (Figure 2, left), both were together erroneously published under the name *C. pectinata* in the Russian monograph by Skrjabin et al. [(15), p. 321], using Ransom's bovid nematode measurements. No original measurements of the deer parasites were published in the Russian monographs (15, 22), so numerical data can only be estimated from the figures.

Unfortunately, the two drawings of male *Cooperia* spicules from bovids and deer (Figure 2), however different they may be, have been widely used to represent alternative characteristics of *C. pectinata*, and they seem to have been mistakenly thought to be its morphotypes.

In summary, it is apparent that deer *Cooperia* differ morphologically from *C. pectinata* (typically found in bovids), to which they have hitherto been assigned for a long time. The aim of the present study was to assess the phylogenetic position of deer parasites in the genus *Cooperia* and to update the morphological and molecular characteristics of *Cooperia* sp. parasitizing red deer (*Cervus elaphus*) or sika deer (*Cervus nippon*) from the Czech Republic. Subsequent aims will involve taxonomic anchoring of the redefined taxon in the context of the current zoological nomenclature rules (25).

## 2 Materials and methods

### 2.1 Collection of nematodes

In 2018–2022, 25 red deer (*Cervus elaphus*)−10 males (M) and 15 females (F) and 30 sika deer (*Cervus nippon*) (10 M + 15 F) were caught in nine official hunts across the Czech Republic. Deer that tested positive for *Cooperia* nematodes were relatively rare, being found in only two regions of northern Bohemia: the Doupov Mountains (Valeč and Doupov hunting regions), and Mimon (the Ralsko hunting ground).

Freshly discarded deer entrails were transported to the Czech University of Life Sciences, Prague (Czech Republic). They were parasitologically examined either immediately or after temporary freezing. For the detection of *Cooperia*, the contents of the small intestine were thoroughly removed, placed on a fine sieve, and washed with running tap water. The residues left on the sieve were transferred to a saline solution on a Petri dish and examined under a stereomicroscope.

### 2.2 Processing of nematodes for morphological and molecular analyses

The collected nematodes were numbered, sexed, and preserved in 70% ethanol. Specimens used for morphological analyses were cleared in a glycerol–ethanol solution by evaporation of the ethanol, and then mounted on glass slides with a 50% glycerol solution and measured using an optical microscope (BX51 light microscope, Olympus). Additional specimens were cleared in a lactophenol solution (26) and then photographed and measured using Quick PHOTO MICRO 3.0 software (Promicra). Special attention was paid to the morphological parameters of the male spicules.

The specimens used for molecular analysis were divided into three body parts: cephalic, middle, and caudal. Only the middle parts were used for molecular analyses, with the cephalic and caudal parts being used for morphological analyses.

### 2.3 Deposited material

The collected specimens were deposited at the Faculty of Agrobiology, Food, and Natural Resources of the Czech University of Life Sciences, Prague (Czech Republic). Additionally, four collected specimens (two males and two females) were deposited in the “Vermes (worm-like animals)” museum collection in the Museum für Naturkunde of the Leibniz Institute for Evolution and Biodiversity (Berlin, Germany).

### 2.4 Molecular analyses

Total DNA was extracted using a QIAamp DNA Mini Kit (Qiagen) using spin column purification according to the manufacturer's protocol. A partial mitochondrial gene sequence (cytochrome oxidase subunit 2, *cox* 2) and a nuclear segment of rDNA (ITS1-5.8S-ITS2) were amplified by PCR.

*Cox* 2 was amplified by PCR as described by Ramünke et al. (6) with slight modifications. The 25-μl PCR mixture contained 1.6 U Top-Bio Taq DNA Polymerase, PCR Blue Buffer, and 1.2 μM of each primer [COII_deg_for (5′-ATKGARTAYCARTTTGGIGGARTT-3′) and COII deg_rev (5′-CTRTGRTTIGCICCRCARATYTC-3′)]. The cycling conditions were as follows: initial denaturation at 95°C for 2 min, followed by 40 cycles of denaturation at 95°C for 15 s, annealing at 49°C for 20 s, and extension at 68°C for 30 s.

The ITS1-5.8S-ITS2 region was amplified by PCR using a 25-μl PCR mixture prepared as described by Callejón et al. (27) with the exception that the following primers were used: forward primer NC5 (5′-GTAGGTGAACCTGCGGAAGGATCATT-3′) and reverse primer NC2 (5′-TTAGTTTCTTTCCTCCGCT-3′) (28), corresponding to the conserved 3′-5′ ends of the ITS1-5.8S-ITS2 region flanking the 18S and 28S regions. The cycling conditions were as follows: 94°C for 3 min, 35 cycles of 94°C for 1 min, 55°C for 1 min, and 72°C for 1 min, followed by a single period at 72°C for 10 min.

The obtained sequences were compared with those of the most closely related species published by Ramünke et al. (6) along with *C. oncophora* from Australian sheep (GQ888713) (29) and *Cooperia* sp. from China (KY769271.1) (30). The trichostrongylid species *Haemonchus contortus* (EU346694.2) and *Teladorsagia circumcincta* (KT428386) were used as outgroups.

The sequences were visually inspected for the accuracy of base calls and the presence of potential heterozygotes. Homologous sequences were aligned using the ClustalW program with the default settings in BioEdit (31). The presence of stop codons was checked using the MEGA software (32).

To estimate the phylogenetic relationships of *Cooperia* spp. with respect to the divergence of *Cooperia oncophora*, Bayesian inference using StarBEAST2 (33) implemented in BEAST v2.7.4 was utilized (34). A phylogenetic analysis was run separately for each partial gene sequence (ITS1-5.8S-ITS2 and *cox 2*) and both simultaneously, generating three independent phylogenetic trees.

Sequence substitution models were estimated in W-IQ-Tree (35) and fitted to a Hasekawa-Kishino-Yano 1985 (HKY85) model with gamma distribution in four categories. The trees were reconstructed under the Strict clock and the Yule model of coalescent evolution. The log files of the three independent runs with 5 × 10^7^ iterations were checked for convergence in Tracer v1.7.2 (36) with 10% burn-in. Combined and annotated trees were graphically generated in FigTree v1.4.4 (http://tree.bio.ed.ac.uk/software/figtree/).

## 3 Results

### 3.1 Occurrence of *Cooperia* in deer in the Czech Republic

Despite the relatively high number of deer examined from various parts of the Czech Republic in 2018–2022 (116 deer belonging to seven ruminant species, not shown), *Cooperia* nematodes occurred occasionally. They were present in only three out of 25 red deer (prevalence = 12.0%) and four out of 30 sika deer (prevalence = 13.3%) and were found only in two regions of northern Bohemia (Table 1).

**Table 1:** Occurrence of *Cooperia* nematodes in seven infected deer in two Czech localities.

**Examined deer**	**Locality**	**No. (sex) of examined deer**	**Sex/age (year) of individual positive deer**	**No. (sex) of *Cooperia* worms**
Red deer (*Cervus elaphus*)	Mimoň	2 (2 F)	F/2	141 (70 M + 71 F)
	Doupov (Valeč)	17 (7 M + 10 F)	F/4	11 (4 M + 7 F)
	Doupov (Valeč)		F/3	4 (1 M + 3 F)
Sika deer (*Cervus nippon*)	Doupov (Valeč)	20 (6 M + 14 F)	F/1	3 (2 M + 1 F)
	Doupov (Valeč)		F/2	3 (1 M + 2 F)
	Doupov (Valeč)		M/6	24 (9 M + 15 F)
	Doupov (Valeč)		F/1	16 (6 M + 10 F)

M, male; F, female for both deer and worms.

The first site (50°39′32^′′^ S, 14°43′29^′′^ E) was Mimon (the Ralsko hunting ground). One red deer was positive out of the two examined (prevalence = 50%). The intensity of nematode infection was 141 worms.

The second site (50°10′30^′′^ S, 13°2′48^′′^ E) was the hunting ground in the Doupov Mountains directly adjacent to the territory around the village of Valeč. Two red deer were positive out of the 17 examined (prevalence = 11.8%) and they had four and 11 worms, respectively. Four sika deer were positive out of the 20 examined (prevalence = 20%), and the intensity of infection ranged from three to 24 worms.

### 3.2 Bayesian phylogenetic trees based on two partial gene sequences

**Figure 3:**
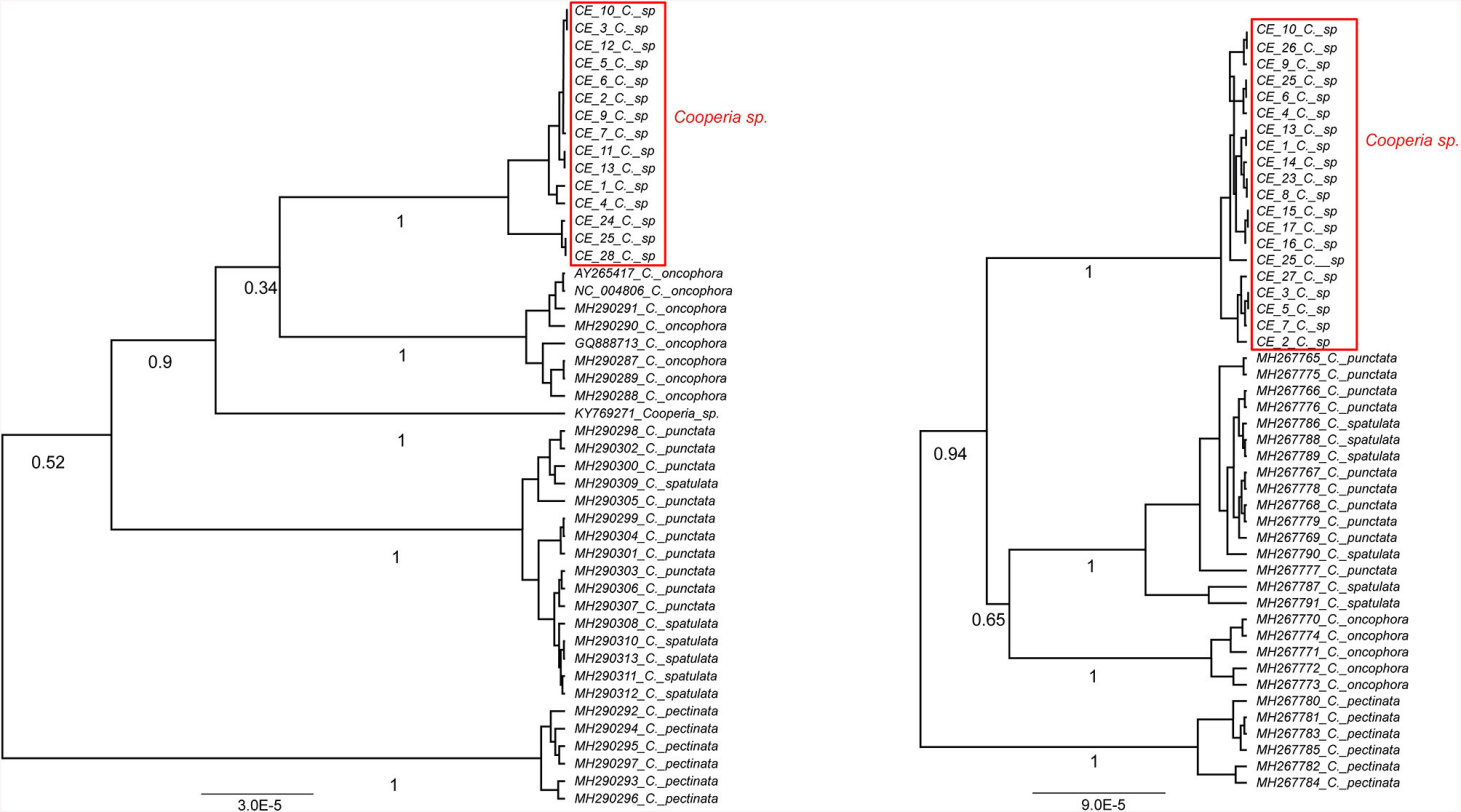
Ultrametric Bayesian phylogenetic trees of *Cooperia* spp. based on the *cox2* gene **(left)** and the ITS1-5.8S-ITS2 region **(right)** of *Cooperia* spp. Branch support values (posterior probability) are displayed under the branches. The trees were rooted with *Teladorsagia circumcincta* (KT428386) and *Haemonchus contortus* (EU346694.2) (not shown), respectively. “*C*” stands for *Cooperia*. Congruently, both trees show *Cooperia* sp. to be an independent lineage with a branch support value of 100%.

**Figure 4:**
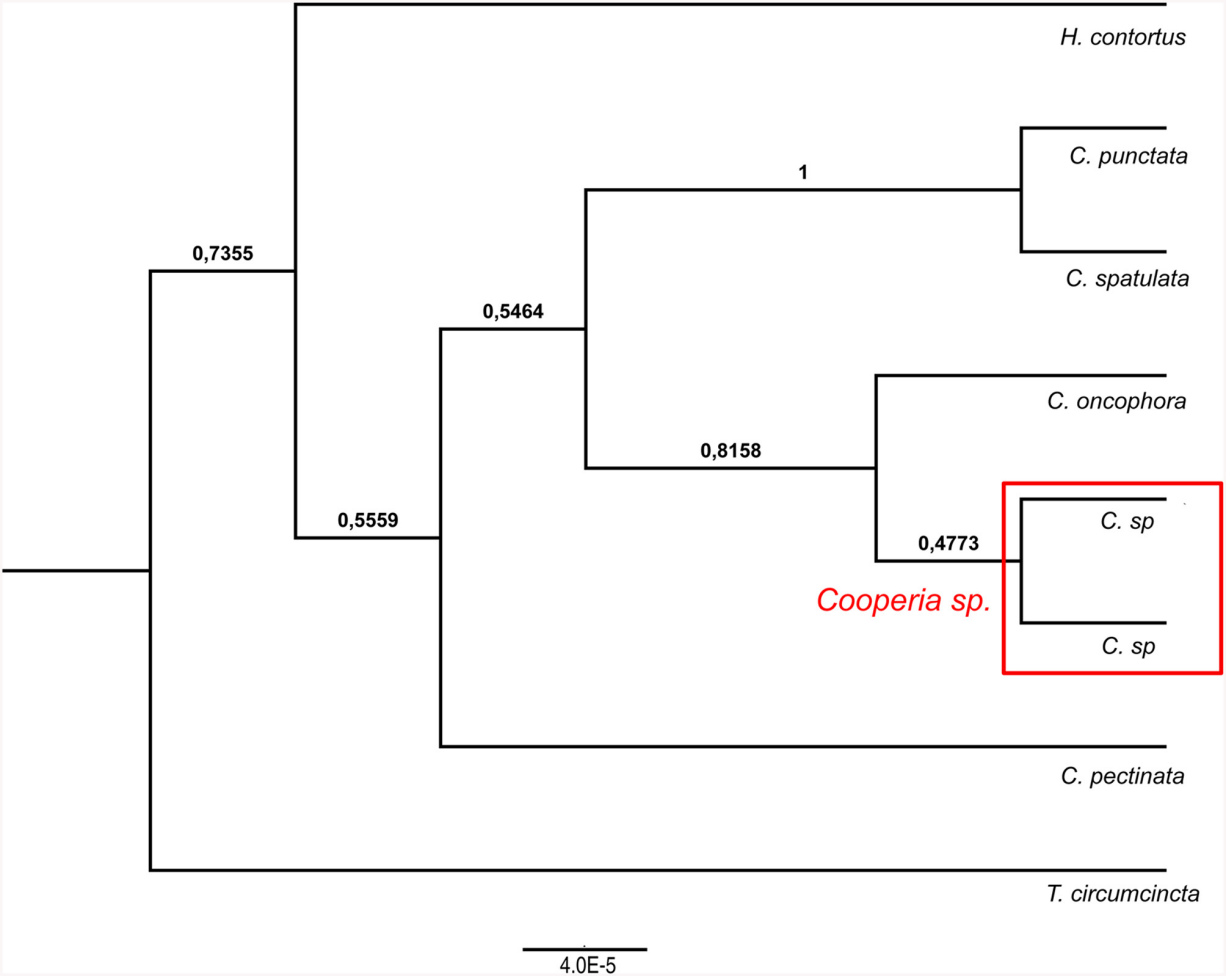
Ultrametric Bayesian phylogenetic tree of *Cooperia* spp. based on combined analysis of mitochondrial DNA (*cox*2) and nuclear ITS1-5.8S-ITS2 rDNA (ITS region). Branch support values (posterior probability) are displayed above the branches. The tree was rooted with *Haemonchus contortus* (EU346694.2) and *Teladorsagia circumcincta* (KT428386). “*C”* stands for *Cooperia*.

The phylogenetic analysis of *Cooperia* sp. from Czech deer based on 18 sequences of *cox* 2 and 20 sequences of the ITS1-5.8S-ITS2 region resulted in six haplotypes (accession numbers OR879242-7) and three haplotypes (accession numbers OR804235, OR804236, and OR804237), respectively. Our data were compared with published data, mainly by Ramünke et al. (6), who compared *C. pectinata, C. punctata, C. spatulata*, and *C. oncophora* and also indicated that *C. spatulata* is most likely only a morphotype of *C. punctata* and its name should be considered a synonym (Figures 3, 4).

Our Bayesian phylogenetic analyses indicated that *Cooperia* sp. from the Czech deer represents a new lineage. The *cox* 2 phylogenetic tree indicated the clustering of this new lineage in the clade containing *C. oncophora*, despite the low branch support (Figure 3, left). The ITS1-5.8S-ITS2 phylogenetic tree showed that this new lineage represents a sister lineage to *C. punctata/spatulata, C. oncophora*. Finally, the *C. pectinata* branch represents the sister lineage to the common cluster described above (Figure 3, right). Also, the phylogenetic tree based on both loci (ITS1-5.8S-ITS2 region and *cox* 2) (Figure 4) agreed with the *cox* 2 phylogenetic tree. Thus, the lineage of *Cooperia* sp. from Czech deer constitutes a new sister lineage to *C. oncophora* and this common branch is a sister lineage to *C. punctata/spatulata*. *Cooperia pectinata* represents the sister lineage to the above cluster.

In conclusion, the Bayesian phylogenetic analysis of combined mitochondrial and nuclear markers (*cox*2 and ITS1-5.8S-ITS2 region) supported the existence of a new independent lineage of *Cooperia* sp. from Czech deer. This analysis confirmed that specimens parasitizing deer game represent a sister lineage to *C. oncophora* while the congener *C. pectinata* is more distantly related. These results indicate a high probability that *Cooperia* sp. that parasitizes deer game does not belong to the *C. pectinata* species that parasitizes bovids.

### 3.3 Morphological description

The measurements of *Cooperia* sp. parasitizing deer are expressed in micrometers (μm) unless otherwise noted, based on 30 males and 30 females.

**Figure 5:**
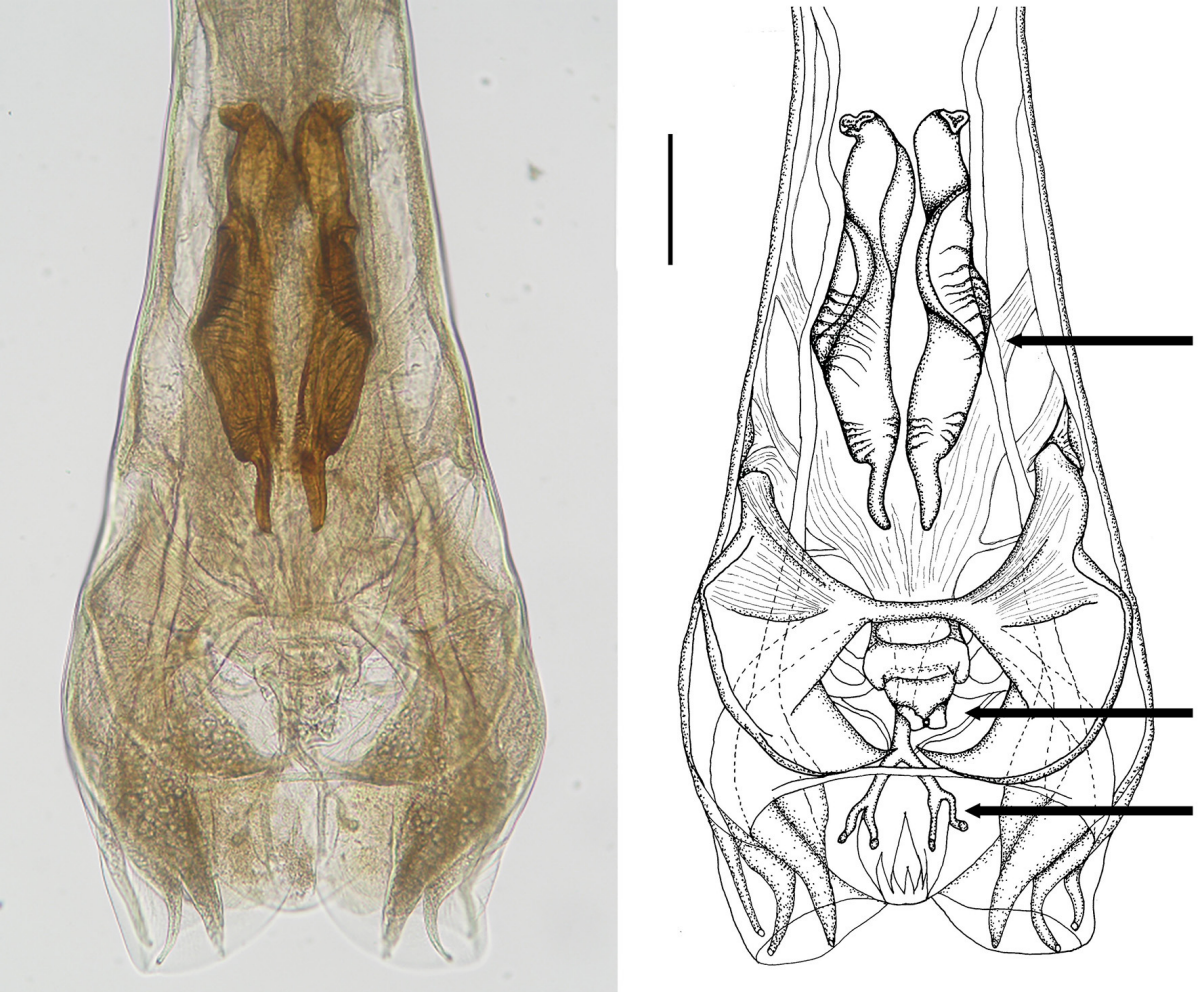
Male genital bursa of deer *Cooperia* sp. Photomicrograph **(left)** and drawing **(right)** outlining spicules (up arrow), genital cone (middle arrow), and dorsal ray (down arrow). Scale bar = 50 μm.

**Figure 6:**
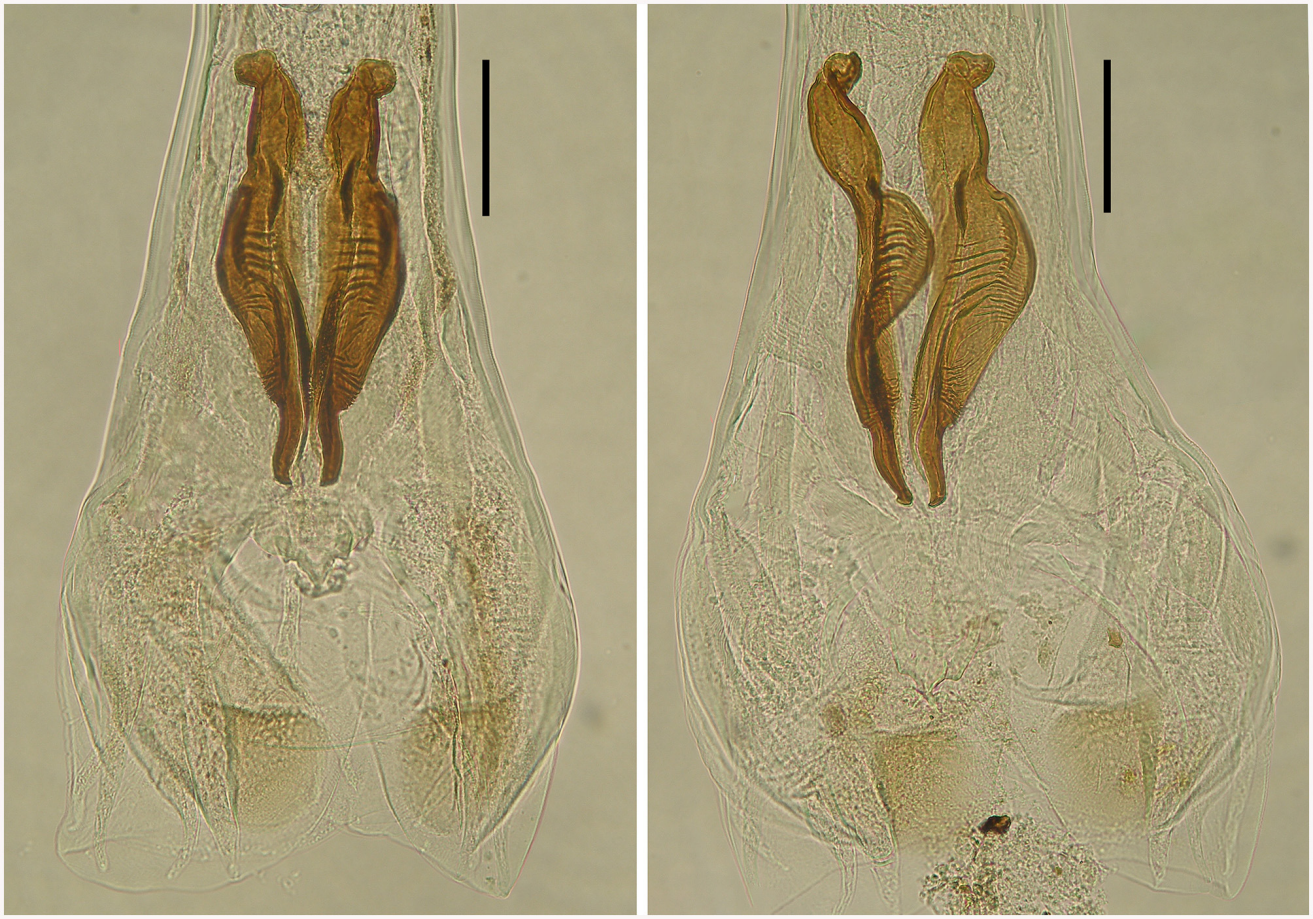
Spicules of two male deer *Cooperia* sp. in dorsal position **(left)** and lateral position **(right)**. Scale bar =100 μm.

Male: Body 5.98–10.24 mm long, 117–189 wide just anterior to bursa, head diameter 32–40, cephalic vesicle up to 105 wide, esophagus 380–515. Bursa 277–400 wide, spicules 265–348 long, 67 maximum spicule width, with four parts (length × width): “short head” 19 × 32, “barrel neck” 62 × 40, “bulky belly” 174 × 67, and “thin tail” 53 × 18. The butterfly-shaped genital cone is situated in the middle of the bursa, 64–222 behind the posterior end of the spicules. The next important morphological characteristics are the shape and size of the dorsal ray of the male bursa: it is a double-branched fork with a total length of 180–208 (196 on average), with the main bifurcation at 56% of the total length (Figures 5, 6). The number of ventrally oriented rays is six on the left and six on the right side, while four are always long and the remaining two are shorter.

Female: Body 7.57–12.74 mm long, its widest part reaching 190–255 behind the vulva, head diameter 33–42, cephalic vesicle 70–77 wide, esophagus 342–622. Vulva opens 2.11–2.70 mm from the posterior end. Anus opens 172–217 from the body end, tail 43–50 wide at the anus level, narrowing sharply to the terminal tip. Eggs in the uterus are 55–61 long and 21–30 wide.

## 4 Discussion

There are no significant differences in the majority of the morphological characteristics between *Cooperia* sp. parasitizing deer (this paper) and *C. pectinata* parasitizing bovids (20), especially when larger sets of individuals are measured and compared. The original description of *C. pectinata* by Ransom (20) was as follows: “Measurements based on few specimens. Male: Body about 7 mm long, by 130–160 μm wide at base of the bursa. Head about 32 μm or more if cuticle is expanded. Esophagus 400 μm long. Dorsal ray including its terminal branches at least 180 μm long. Spicules 240–280 μm long, distal third much more slender than remainder, middle third with a prominent curved ventrally-projecting edge corrugated on the inner surface. Female: 7.5–9 mm long, 110–135 μm wide close to the vulva. Head 35–50 μm wide, esophagus 360–400 μm long. Posterior body end gradually attenuated backward, terminating in a slender sharply pointed tail. Vulva opens 1.6–2 mm from the tip of the tail, its opening with projecting vesicular lips. Anus about 175 μm from the body end. Eggs 70–80 μm long and 36 μm wide.” **[sic]** Moreover, Gibbons (16) provided figures of the dorsal ray of *C. pectinata* from impalas in Africa (original Figure 78), which measured 269 μm in total, and its proximal unbranched part represented 45% of its length.

Although deer and bovid *Cooperia* females do not exhibit significant differences, two male species-specific characteristics clearly distinguish these lineages: the morphology of the spicules and, to some extent, the shape of the dorsal ray of the male bursa (37).

First, spicular morphology differs between the *Cooperia* lineages of bovids and deer (though the length and width of the spicules are comparable between the two lineages). Both lineages have four morphologically distinctive parts, typical of the genus *Cooperia*: a short anterior head, a barrel neck, a noticeable central edge with well-demarcated corrugations (a bulky belly), and a thin tail. The species-specific characteristic lies in the length of the distal thin tail part, which is significantly longer in bovid *C. pectinata* (one third of the total spicule length according to the original Figure 1, by Ransom (19) or Figure 2, left, by Baylis (21), while this thin tail part represents less than one fifth (~14%) in deer *Cooperia* sp. (Figures 2, right, 5, 6).

Second, the dorsal ray of the male bursa differs slightly in shape. Its total length is similar between bovid and deer *Cooperia*, more than 180 μm in bovids according to Ransom (21) and 180–208 in deer (this work), and it is twice forked in its posterior half. However, the main bifurcation is located before half of its total length (~45%) in bovid *C. pectinata* (16) but closer to the end of the bursa at 56–60% of the total ray length, in deer *Cooperia* sp. (Figure 5).

Nevertheless, these discrepancies have not prevented *Cooperia* from deer from being mistakenly identified as *C. pectinata* in various parts of Europe, such as the Czech Republic (38), most likely Austria (39, 40) Norway (41), and certainly New Zealand (42). This was mainly caused by the dubious species characteristics published in the key monograph by Skrjabin et al. (15), with the subsequent widespread acceptance of this erroneous information in the past (15, 22, 38) and more recently (39–42).

The overall morphological results clearly confirmed the results of our phylogenetic analysis. It is therefore certain that the *Cooperia* sp. parasitizing the Czech deer does not belong to the species *C. pectinata*.

### 4.1 History of deer *Cooperia* spp.

Our molecular and morphological characterization of the nematodes from Czech deer led to a review of historical information on deer nematodes. The first scientific description of a strongylid species from a deer, caught near Greifswald (Germany) was provided by Rudolphi (43), who used the name *Strongylus ventricosus*. Over the years, this ancient deer nematode has been reassigned twice (as *C. curticei* or *C. oncophora*) to the newer genus *Cooperia* Ransom, 1907. However, both suggestions are currently invalid (17).

Original description of *Strongylus ventricosus* Rudolphi, 1809 (translation from Latin, https://www.biodiversitylibrary.org/item/50353#page/5/mode/1up), [(43), p. 222]:

“*Strongylus ventricosus*, R.”

“*Strongylus*: with a thin, winged head, a male blunt bursa behind, and a female tail awl-shaped.

Hab: four specimens found in the upper part of the intestines of the *Cervus elaphus*, February (1809, current note)

Description: worms six to eight “lines” long, very thin, reddish.

Male: the head is thin and winged by a thin membrane on both sides. The body is thin and almost linear toward the middle, and then it gradually thickens and forms the genital bursa at the end. This is obtuse, radiating, with thinly folded membranes, so that I cannot tell the number of lobes. A thin feeding tube, running through the middle of the body, gives the worm a striated face.

Female: the head as in male, but in another specimen the wing-shaped membrane is wider. The body is linear anteriorly, in the third part of the worm it is initially very thick, as if knotted, then thins again, the tail is awl-shaped. The vulva is partially protruding” [sic].

**Figure 7:**
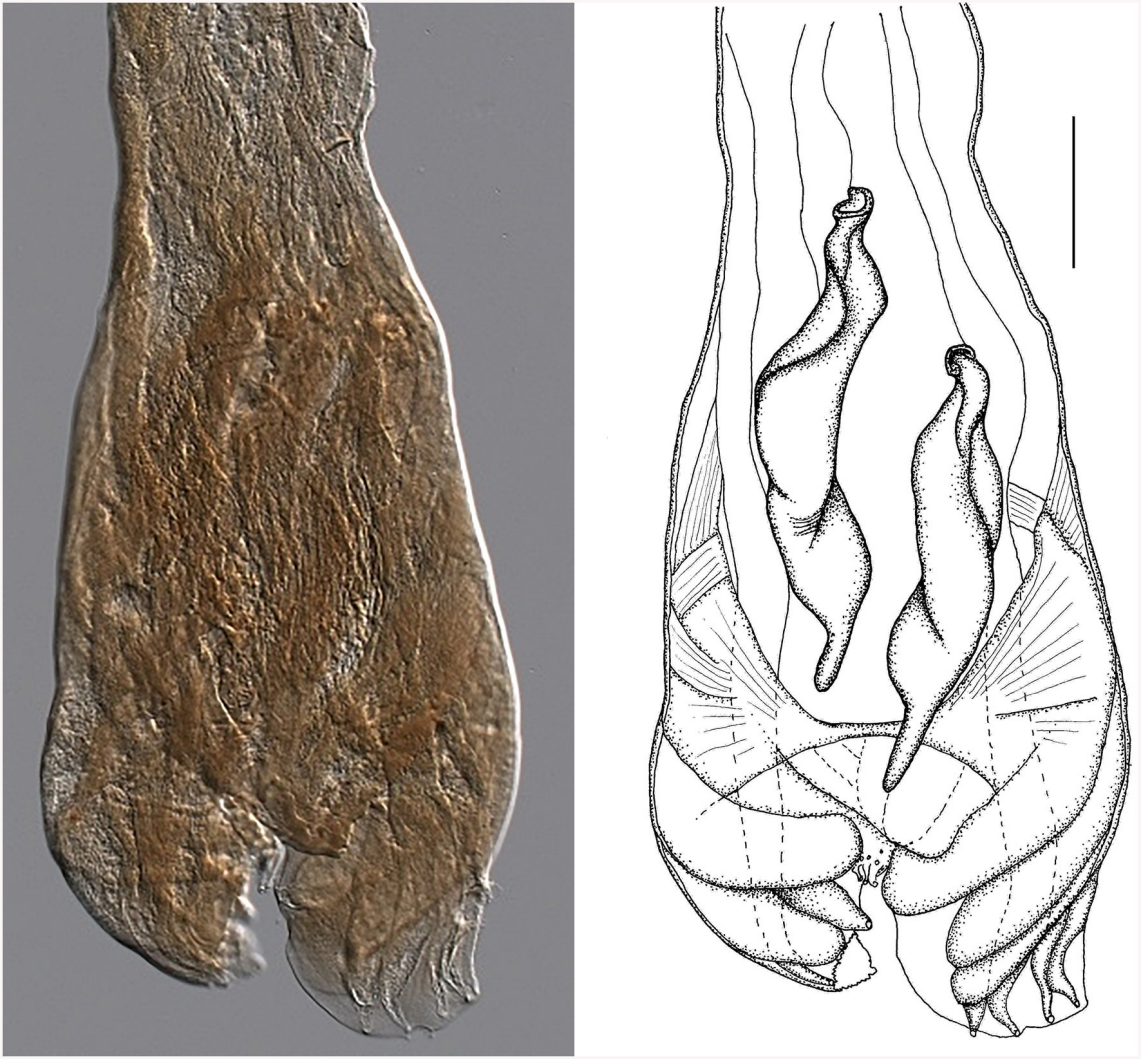
Male genital bursa of the type specimen *Strongylus ventricosus* Rudolphi, 1809 (current reconstruction) from the Museum für Naturkunde (Berlin). Drawing **(left)** and photomicrograph **(right)**. Scale bar = 50 μm.

The type material of the species *S. ventricosus* Rudolphi, 1809, was deposited in the Museum für Naturkunde (Berlin) under no. AHC 49508. It is in the form of permanent slides made from five voucher specimens of deer trichostrongylid parasites (one male and four females). As a final step to clarify the species affiliation of *Cooperia* sp. from Czech deer, our new material was compared to the five voucher specimens of deer trichostrongylid parasites. Unfortunately, all five specimens are in poor condition, and only the male bursa is intact, although it is also considerably damaged (Figure 7).

Morphology of the bursa and spicules of the type specimen *Strongylus ventricosus* Rudolphi, 1809 (measurements in mm from Figure 7): bursa 306 wide, spicules 308 long on average, maximum spicule width 61, Each spicule has four parts: short head, two middle parts (barrel neck and bulky belly) which are difficult to distinguish, and clearly visible characteristic thin tail 51 long on average which represents 16.5% of the spicule length.

Although all structures are quite damaged, the size of the spicules matches well with *Cooperia* sp. males from Czech deer (in the Morphological description, subsection of the Results sections). Some of the slightly larger dimensions of the spicule parts were probably caused by flattening during the preparation of permanent slides of the museum material. The data from the original description of *S. ventricosus* also concur with the morphology of *Cooperia* sp. from Czech deer.

As there is a good agreement between our new material (*Cooperia* sp. from Czech deer) and the voucher specimens of the deer trichostrongylid parasite *S. ventricosus*, which were registered in the Museum für Naturkunde (Berlin), the transfer of the ancient species *S. ventricosus* Rudolphi, 1809, into the genus *Cooperia*, and the creation of the species *Cooperia ventricosa* comb. nov. should be seriously considered. The ideal solution to the problem would be to obtain new material for nematodes of the genus *Cooperia* from the type host *Cervus elaphus* from the type locality near Greifswald (Germany), and to redescribe *C. ventricosa* according to the stricter ICZN rules (25).

**Taxonomic summary of** ***Cooperia ventricosa* (Rudolphi, 1809) comb. nov. (****Figures 5****–****7****) from deer game**

Class Chromadoria (Order Rhabditina, Superfamily Strongyloidea, Family Trichostrongylidae, Tribe Cooperiinii, genus *Cooperia* Ransom, 1907) (44, 45).

***Cooperia ventricosa* (Rudolphi, 1809), comb. nov**.

***Synonym:*** *Strongylus ventricosus* Rudolphi, 1809.

***Type host***: red deer *Cervus elaphus* Linnaeus, 1758 (Artiodactyla: Cervidae).

***Other hosts***: European fallow deer *Dama dama* (Linnaeus, 1758), sika deer *Cervus nippon* Temninck, 1838.

***Site of infection:*** small intestine.

***Type locality***: vicinity of Greifswald, Germany (40).

***Documented distribution*:** various regions of Europe (38, 46), New Zealand (42), northern regions of the Czech Republic—new geographical record (this paper).

***Type material*:** Museum für Naturkunde Berlin, collection “Vermes,” catalog Entozoa, E.258, 6 syntype fragments in deteriorated condition, mounted as 5 glycerol-paraffin slides on Cobb aluminum frames by B. Neuhaus on 16.XI.2021, E.258-1 female, E.258-3 female, E.258-5 male, sex of E.252-2, and E.252-4 unknown.

***Morphological descriptions***: (43), [(22)—Figure 142], [(15)—Figure 164], this paper

***Remarks:*** A member of the genus *Cooperia* that shares all the morphological characteristics that define the genus *C. ventricosa*, differs from the most similar species *C. pectinata* based on the following features: the shape of the male spicules and that of the dorsal ray of the male genital bursa.

## 5 Conclusion

In conclusion, this integrative study of the ruminant parasite of the genus *Cooperia* (Nematoda, Trichostrongyloidea) revealed the existence of a separate species found in red and sika deer. According to the morphology, it was quite similar to, but not identical to, *Cooperia pectinata*, which was long erroneously considered to be a parasite of both bovids and deer game. A new comparative analysis of *cox*2 and ITS rDNA partial gene sequences from a spectrum of *Cooperia* spp. revealed that this nematode represents a separate lineage, morphologically nearly identical to the ancient deer nematode *Strongylus ventricosus* Rudolphi, 1809. We, therefore, suggest its resurrection as *Cooperia ventricosa* (Rudolphi, 1809) comb. nov., which should ideally be followed by verification by collecting and analyzing new nematode material from the type deer host and the type locality near Greifswald.

## Data availability statement

The datasets presented in this study can be found in online repositories. The names of the repository/repositories and accession number(s) can be found below: https://www.ncbi.nlm.nih.gov/genbank/, OR804235, OR804236, and OR804237.

## Ethics statement

The animal studies were approved by Ethical Committee of Czech University of Life Sciences Prague. The studies were conducted in accordance with the local legislation and institutional requirements. Written informed consent was not obtained from the owners for the participation of their animals in this study because the game deer owners did not require written informed consent.

## Author contributions

MA: Conceptualization, Data curation, Investigation, Writing – original draft. EK: Conceptualization, Data curation, Methodology, Writing – original draft, Writing – review & editing. IL: Conceptualization, Funding acquisition, Methodology, Project administration, Resources, Writing – review & editing. VH: Data curation, Investigation, Resources, Supervision, Writing – review & editing. BN: Data curation, Methodology, Resources, Supervision, Validation, Writing – review & editing. IJ: Conceptualization, Investigation, Supervision, Writing – review & editing. MP: Methodology, Visualization, Writing – review & editing. JM: Data curation, Formal analysis, Investigation, Methodology, Validation, Visualization, Writing – review & editing. MŠ: Conceptualization, Data curation, Supervision, Validation, Writing – original draft, Writing – review & editing.
