# Corrigendum: A revision of the trichostrongylid nematode *Cooperia* Ransom, 1907, from deer game: recent integrative research confirms the existence of the ancient host-specific species *Cooperia ventricosa* (Rudolphi, 1809)

**DOI:** 10.3389/fvets.2024.1388292

**Published:** 2024-02-29

**Authors:** Martina Albrechtová, Eva Štefková Kašparová, Iva Langrová, Vlastimil Hart, Birger Neuhaus, Ivana Jankovská, Miroslav Petrtýl, Jan Magdálek, Marta Špakulová

**Affiliations:** ^1^Department of Zoology and Fisheries, Faculty of Agrobiology, Food and Natural Resources, Czech University of Life Science Prague, Prague, Czechia; ^2^Department of Game Management and Wildlife Biology, Faculty of Forestry and Wood Sciences, Czech University of Life Science Prague, Prague, Czechia; ^3^Museum für Naturkunde, Leibniz Institute for Evolution and Biodiversity, Berlin, Germany; ^4^Institute of Parasitology, Slovak Academy of Sciences, Košice, Slovakia

**Keywords:** *Cooperia ventricosa*, *Cooperia pectinata*, deer game, mitochondrial DNA, ribosomal DNA, redescription, gastrointestinal nematodes

In the published article, there was an error in the description of type material in the taxonomic summary of Cooperia ventricosa (Rudolphi, 1809) comb. nov. from deer game.

A correction has been made to Discussion, *History of deer Cooperia spp*., paragraph 22. The sentence previously stated:

“Museum für Naturkunde (Berlin), AHC 49508 (holotype), vouchers (two males and two females).”

The corrected sentence appears below:

“Museum für Naturkunde Berlin, collection “Vermes,” catalog Entozoa, E.258, 6 syntype fragments in deteriorated condition, mounted as 5 glycerol-paraffin slides on Cobb aluminum frames by B. Neuhaus on 16.XI.2021, E.258-1 female, E.258-3 female, E.258-5 male, sex of E.252-2, and E.252-4 unknown.”

The authors apologize for this error and state that this does not change the scientific conclusions of the article in any way. The original article has been updated.

